# Antenatal spectrum of congenital heart disease: Experience from a Northern Indian referral unit

**DOI:** 10.6026/973206300212432

**Published:** 2025-08-31

**Authors:** Reema Bhatt, Raja Rajeswari Venkatesh, Gaurav Juneja, Siddharth Tewari

**Affiliations:** 1 Department of Fetal Medicine, Amrita Institute of Medical Sciences, Faridabad, Delhi NCR, India

**Keywords:** Congenital heart defects, prenatal screening, fetal echocardiography, color Doppler, maternal risk factors, genetic testing

## Abstract

Congenital heart defects (CHDs) remain the most common birth anomalies, yet many cases are still detected late despite fetal ultrasound advances. This retrospective study analysed 3,660 antenatal scans, diagnosing 120 CHD cases, with Ventricular Septal Defect being most common (55.8%), followed by Tetralogy of Fallot (15%). A strong association with maternal risk factors and extra cardiac anomalies was noted, stressing the need for complete fetal evaluation. Thus, strengthening second-trimester screening, clinician training and use of genetic testing can improve early CHD detection and outcomes.

## Background:

Congenital heart defects (CHDs) are the most common birth anomalies, affecting nearly 1% of live births worldwide [1]. Fetal cardiology has progressed greatly since the 1980s, with fetal echocardiography now a key tool for prenatal CHD detection [2]. However, detection rates still vary between 15% to 55%, depending on the anomaly, examiner expertise and gestational age at screening [3]. The second trimester (18-22 weeks) is considered ideal for fetal cardiac evaluation, as heart structures are better developed [4]. With improved ultrasound technology, some anomalies can now be detected as early as the first trimester [5]. The effect of prenatal CHD detection on mortality remains debated. While tertiary centres report better survival with early diagnosis in severe cases like hypo plastic left heart syndrome and transposition of great arteries, large population studies show inconsistent outcomes [6, 7]. Regardless the prenatal CHD detection remains vital for pregnancy management, parental counselling, delivery planning and reducing postnatal complications [8]. Higher detection rates of severe CHDs have also led to increased pregnancy terminations, altering CHD epidemiology in some regions [9]. Despite technological advances, prenatal CHD detection still shows gaps. Detection rates vary across centres due to sonographer expertise and resources. Accurate diagnosis requires proper training and strict protocols. Therefore, it is of interest to evaluate current prenatal CHD detection, focusing on accuracy, gaps and perinatal outcomes. It adds to existing data by assessing detection trends and prenatal pick-up rates.

## Materials and Methods:

This retrospective observational study was conducted at a tertiary fetal medicine centre over one year (June 2023 to May 2024) to assess antenatal diagnosis of congenital heart defects (CHDs) using fetal echocardiography and anomaly scans. A total of 3,660 pregnant women underwent ultrasound screening-950 in first trimester (26%), 1,150 in second (31.4%) and 1,500 in third trimester (41%). Referrals were routine or based on abnormal four-chamber views, suspected anomalies, maternal diabetes, autoimmune conditions or family history of CHDs. Scans were done using GE Voluson E10 as per ISUOG guidelines, including four-chamber view, outflow tract assessment, colour Doppler and rhythm evaluation for structural and conduction defects. CHDs were classified as septal defects, cyanotic heart disease, outflow anomalies and conduction issues like bradycardia with reduced AV conductivity. Trimester-wise detection was recorded. All CHD cases were screened for extra-cardiac anomalies like craniofacial, brain or skeletal defects. Maternal risk factors such as GDM, autoimmune disorders and FGR were documented. Genetic testing (karyotyping, CMA) was offered in selected severe or syndromic cases. Data were analysed using SPSS version 26 from electronic records. Institutional ethics approval was obtained, no extra procedures beyond routine care were done and informed consent was taken for genetic testing.

## Results:

A total of 3,660 antenatal scans were done, maximum in the third trimester (41%), followed by second (31.4%) and first trimester (26%) (Table 1 - see PDF). This indicates late scans improve visibility, but early detection is still essential. Among 120 CHD cases, VSD was most common (55.8%), followed by Tetralogy of Fallot (15%) and Complex Cardiac Disease (6.7%) (Table 2 - see PDF, Figure 1 - see PDF). Tricuspid Regurgitation appeared early, while Congestive Cardiac Failure was detected only later, highlighting the need for serial fetal echocardiography. Extra-cardiac anomalies were seen in 10%, like Hypoplastic Nasal Bone with Tricuspid Regurgitation and Vermian Hypoplasia with VSD+DORV (Table 3 - see PDF), showing the importance of complete fetal assessment. Maternal and fetal factors were seen in 45% of CHDs (Table 4 - see PDF). Bradycardia was linked to maternal Anti-RO/LA positivity, FGR with HLHS and Complex CHD and VSD with GDM, stressing the role of maternal risk screening. VSD (40%) was most frequent, followed by Tetralogy of Fallot (10%), Complex CHD (7%) and other defects like DORV, ASD, AVSD, HLHS, Bradycardia, each contributing 5% (Table 5 - see PDF). Rare anomalies like Three Chamber Heart and Double Aortic Arch were noted, needing specialised evaluation.

## Discussion:

The results show many CHDs were missed early and detected later, highlighting the need for second and third-trimester scans. Tricuspid Regurgitation appeared early, but serious defects like Congestive Cardiac Failure were seen later. Relying on a single scan risks missing defects; serial scans improve detection. CHDs often coexist with extra cardiac anomalies and maternal risks, making complete fetal-maternal assessment essential. Genetic testing, though underused, detected chromosomal abnormalities in nearly half the cases. Scan distribution raises concern-41% of scans were done in the third trimester, 31.4% in the second and only 26% in the first. Late scans improve anatomical visibility but delay diagnosis, risking missed interventions. Early detection of major CHDs like transposition of great arteries between 18-22 weeks can reduce neonatal deaths by nearly 30% [10]. Second trimester remains ideal for detecting VSDs and outflow tract anomalies, yet only 31.4% scans were done during this window [4]. First-trimester screening offers 67.7% sensitivity in high-risk cases, but minor defects often go unnoticed [11]. Third-trimester scans help detect late-onset anomalies, but early diagnosis provides better outcomes, reducing preoperative instability by 50% and detecting up to 85% of major CHDs [11, 12 and 13]. Serial fetal echocardiography throughout pregnancy remains is essential [12]. Ventricular Septal Defect (VSD) was the most common CHD (55.8%, 67 cases), with larger VSDs (>3mm) leading among major defects, followed by endocardial cushion defects, stressing the need for careful ventricular septum evaluation [14]. Tetralogy of Fallot (19 cases) was also significant, highlighting the role of prenatal detection for better postnatal care. Most CHDs were picked during second trimester, supporting routine screening at 18-22 weeks [15]. All pregnancies should undergo anomaly scans, with fetal echocardiography reserved for abnormal or high-risk cases [14, 15]. Detection rates vary widely-only 9% for TAPVC but up to 67% for HLHS in U.S. studies [16, 17] and 63% HLHS detection reported from Sweden, showing global gaps [18]. Many CHDs were still diagnosed late in the third trimester, often milder cases. Though early detection is ideal, some defects develop later [19]. As most CHDs occur in low-risk pregnancies, targeted fetal echo should be based on abnormal scans or specific risk factors [20]. Prenatal ultrasound shows high diagnostic accuracy-90.5-91.66% for complex CHDs and up to 98.6% for simple ones. Yet, there is scope to further improve imaging techniques [21]. This study supports routine CHD screening but also highlights existing gaps. The variation in CHD types and detection timing justifies a multi trimester screening approach to enhance detection rates and optimise perinatal outcomes.

The link between CHDs and extra cardiac anomalies is well known; as such defects increase surgical risk, complications and mortality [22]. In this study, 10% of CHD cases had extra cardiac anomalies, mainly craniofacial, CNS, skeletal (25% each) and renal defects (16.7%), consistent with earlier reports. Fetal echocardiography is vital, especially in high-risk pregnancies [4]. Markers like increased nuchal translucency, hydrops or genetic syndromes need detailed evaluation. 2D imaging, colour Doppler and pulse Doppler improve detection [11]. This study highlights the need to integrate cardiac and extra cardiac screening for better counselling and care. Maternal and fetal factors contribute to CHDs, reflecting complex origins. Bradycardia with reduced AV conductivity was linked to Anti-RO/LA positivity, VSD to GDM and HLHS to fetal growth restriction. These findings align with studies linking autoimmune and metabolic disorders to fetal heart defects. PGD also increases risk of hypertrophic cardiomyopathy due to septal thickening, affecting cardiac function [17]. CHD increases the risk of fetal growth restriction (FGR), especially in operated VSDs and Tetralogy of Fallot, likely due to hypoxia and poor placental perfusion [23]. A large study of over 2 million births reported a 4-fold higher CHD risk in infants of mothers with pre gestational diabetes, mainly affecting outflow tracts, similar across type 1 and 2 diabetes [24]. These findings stress the need for targeted screening and multidisciplinary care in high-risk pregnancies. This study's CHD pattern follows global trends with few regional variations. VSD was most common (55.8%, 67/120), higher than the global average (3.07 vs 2.62 per 1,000), possibly due to better detection or local factors [25, 26]. Tetralogy of Fallot (15%) and complex CHDs (6.7%) matched global data where cyanotic CHDs form nearly 25% of severe cases [27, 28]. Some patterns were notable, Hypo plastic Left Heart Syndrome (HLHS) was rare (1.7%), consistent with global decline from 0.689 to 0.475 per 1,000 births, likely due to better detection and terminations in high-resource countries [29]. Regional CHD differences persist-Asia shows higher rates (9.3/1,000) than Europe (8.2/1,000) and Africa (2.3/1,000), mostly due to diagnostic access and genetic factors [30]. ASDs are more reported in wealthier regions, suggesting under diagnosis elsewhere. Mild CHDs like VSD, ASD and PDA are rising with better second-trimester imaging, contributing to 93.4% of CHD detection [29]. Severe CHDs like LVOTO are declining; matching low HLHS rates here [25, 26- 27]. Underreported regions still show low CHD rates (1.9-2.3/1,000) stressing a need for standard protocols like Indian data. VSD is still the most common CHD diagnosed antenatally in multiple studies [31]. A 20 years Northern Indian study confirmed high CHD burden with VSD leading again [32]. A Kashmir centre also found VSD most frequent then TOF matching our results [33]. Military hospital study reported more antenatal CHD cases likely due to better fetal scan awareness and use [34]. This study reflects both strengths and gaps. Of 3,660 scans, 41% were third trimester, 31.4% second and 26% first trimester. Many CHDs appear later as structures mature delayed diagnosis impacts care and counselling. Improving detection needs stronger focus on second-trimester scans (18-22 weeks), targeted by maternal risk. Sonographer training, serial scans and genetic testing for severe cases are crucial. A multidisciplinary team remains key for better CHD detection and care.

## Conclusion:

Prenatal CHD detection is improving; many cases are still being diagnosed late. Second-trimester scans between 18 to 22 weeks, which are ideal for picking major defects, are not being done consistently in all cases. More focus on maternal risk factors, repeated fetal scans and offering genetic testing wherever needs will definitely help in better detection. A team approach involving fetal medicine experts, cardiologists and neonatologists is very important to improve diagnosis and overall care of these newborns.
